# Effects of Nitrosyl Iron Complexes with Thiol, Phosphate, and Thiosulfate Ligands on Hemoglobin

**DOI:** 10.3390/ijms25137194

**Published:** 2024-06-29

**Authors:** Olga V. Kosmachevskaya, Elvira I. Nasybullina, Olesya V. Pokidova, Natalia A. Sanina, Alexey F. Topunov

**Affiliations:** 1 Bach Institute of Biochemistry, Federal Research Center of Biotechnology, Russian Academy of Sciences, Moscow 119071, Russia; 2 Federal Research Center of Problems of Chemical Physics and Medicinal Chemistry, Russian Academy of Sciences, Moscow Region, Chernogolovka 142432, Russia

**Keywords:** hemoglobin, oxidative modification, peroxidation, *tert*-butyl hydroperoxide, nitrosyl iron complexes, nitric oxide, antioxidant effect

## Abstract

Nitrosyl iron complexes are remarkably multifactorial pharmacological agents. These compounds have been proven to be particularly effective in treating cardiovascular and oncological diseases. We evaluated and compared the antioxidant activity of tetranitrosyl iron complexes (TNICs) with thiosulfate ligands and dinitrosyl iron complexes (DNICs) with glutathione (DNIC-GS) or phosphate (DNIC-PO_4_^−^) ligands in hemoglobin-containing systems. The studied effects included the production of free radical intermediates during hemoglobin (Hb) oxidation by *tert*-butyl hydroperoxide, oxidative modification of Hb, and antioxidant properties of nitrosyl iron complexes. Measuring luminol chemiluminescence revealed that the antioxidant effect of TNICs was higher compared to DNIC-PO_4_^−^. DNIC-GS either did not exhibit antioxidant activity or exerted prooxidant effects at certain concentrations, which might have resulted from thiyl radical formation. TNICs and DNIC-PO_4_^−^ efficiently protected the Hb heme group from decomposition by organic hydroperoxides. DNIC-GS did not exert any protective effects on the heme group; however, it abolished oxoferrylHb generation. TNICs inhibited the formation of Hb multimeric forms more efficiently than DNICs. Thus, TNICs had more pronounced antioxidant activity than DNICs in Hb-containing systems.

## 1. Introduction

Nitric oxide (NO^•^) is a lipophilic radical molecule produced in all organisms during enzymatic and non-enzymatic reactions [1,2]. NO^•^ orchestrates numerous physiological processes such as cell proliferation, apoptosis, neurotransmission, and immune response. The greatest biological effect of NO^•^ arises from its reactions with soluble guanylate cyclase (EC 4.6.1.2). NO^•^ is bound to a receptor (NO receptor) that triggers its guanylate cyclase activity, and this gives rise to a transduction pathway. Other important NO^•^ effects are the actions on hemoproteins, such as hemoglobin (Hb), and cytochromes and extremely rapid reactions with free radicals. One of these reactions leads to the formation of a highly toxic peroxynitrite [2,3]. Therefore, high concentrations (above 1 mM) of NO^•^ exert pathophysiological effects [2,3].

Nitric oxide significantly contributes to the functioning of the cardiovascular system by regulating the tone of small and medium vessels. It displays an anticoagulant effect, suppresses platelet aggregation and leukocyte adhesion, and inhibits the production of vasoconstrictors and the synthesis and oxidation of low-density lipoproteins [4,5,6,7]. A decrease in NO^•^ production and bioavailability causes chronic diseases such as hypertension, ischemic reperfusion injury, atherosclerosis, and diabetes [8,9].

NO^•^ is currently used to regulate blood pressure [6,10], as a cardioprotective mediator upon ischemia, hypertension, and stroke [10,11], and for cancer treatment [12,13]. Since NO^•^ has a short lifetime, nitric oxide donors can be used in medicine, for instance, organic nitrates (RONO_2_), nitrites (RONO), S-nitrosothiols (RSNO), nitrosamines, sydnonymines, N-diazenium diolates (NONOates), and nitrosyl transition metal complexes [1,14,15,16,17]. 

The cardioprotective properties of dinitrosyl iron complexes (DNICs) with thiol-containing ligands (DNIC-RS) have been extensively studied so far [11,18,19]. Unlike organic nitrates, routinely used in medicine as vasodilators, DNICs do not induce tolerance upon long-term use.

Currently, naturally occurring DNICs with glutathione and cysteine ligands as well as their synthetic analogues have been extensively studied [16,19,20,21,22,23]. These complexes are used as models of active nitrosyl Fe-S protein clusters that exist in all living organisms [1,16]. The obtained mono- and binuclear nitrosyl iron complexes with functional sulfur-containing ligands release NO^•^ at a physiological pH without additional activation, exist in a crystalline state, and break down into non-toxic compounds [16]. These properties make these complexes suitable for biomedical practice.

The most studied anionic binuclear tetranitrosyl iron complex (TNIC) is the complex with sodium thiosulfate Na_2_[Fe_2_(S_2_O_3_)_2_(NO)_4_]·4H_2_O. It is an efficient NO^•^ donor [24] and has been shown to exert a therapeutic effect on rat myocardium upon ischemia-reperfusion injury. It also exhibits a vasodilating effect, efficiently restoring the coronary blood flow and reducing the systolic blood pressure. This complex displays antitumor activity in vitro [12,13].

NO^•^ is a molecule with a multidirectional action, which depends on its concentration and redox conditions. NO^•^ is known to possess antioxidant activity [25,26,27,28,29,30,31,32,33,34,35] mediated by its direct interaction with free radicals as well as the activation of signal transmission pathways. Such activation promotes the synthesis of endogenous antioxidants or suppresses the responses to pro-inflammatory stimuli [2,5]. The antioxidant effect of NO^•^ metabolites on Hb has also been shown [30,31,35].

The antioxidant properties of nitrosyl iron complexes are still controversial since the iron catalyzes lipid peroxidation (LPO), thereby exhibiting prooxidant effects. In addition, NO^•^ released from the complexes can also exhibit a prooxidant effect, being a source of peroxynitrite. Therefore, we performed a comparative study of the antioxidant/prooxidant activity of nitrosyl iron complexes with glutathione, phosphate, and thiosulfate ligands in systems that model Hb peroxidation.

## 2. Results

### 2.1. Formation of Free Radical Products during Hemoglobin Oxidation with Tert-Butyl Hydroperoxide 

We studied the influence of DNICs with glutathione (DNIC-GS) and phosphate (DNIC-PO_4_^−^) ligands and the influence of TNIC with thiosulfate ligands on the formation of free radical intermediates using the model of hemoglobin peroxidation by *t*-BOOH. 

The reaction between methemoglobin (metHb) and an organic hydroperoxide results in the generation of oxoferrylHb with a radical on the porphyrin or the protein component as well as alkoxyl and alkylperoxyl hydroperoxide radicals (Reactions (1)–(4)) [25,36]:

Hb-Fe^3+^ + ROOH → Hb-Fe^4+^=O + RO^•^ + H^+^ (1)

Hb-Fe^3+^ + ROOH → Hb^•^-Fe^4+^=O + ROH (2)

Hb^•^-Fe^4+^=O + ROOH → Hb-Fe^4+^=O + ROO^•^ + H^+^ (3)

Hb-Fe^4+^=O + ROOH → Hb-Fe^3+^-OH + ROO^•^ (4)

According to [37], the main free radical generated in this reaction system is the alkoxy (*tert*-butoxy) radical.

Free radicals were registered using luminol chemiluminescence. The interaction of luminol (3-aminophthalhydrazide) with peroxyl radicals produces extremely unstable oxyl radicals of luminol (^•^LH), which decompose to form nitrogen and 3-aminophthalic acid in the excited electronic state (P*). The latter returns to the ground state, emitting a photon, as follows:

ROO^•^ + LH_2_ → ROOH + ^•^LH (5)

^•^LH → P* + N_2_ (6)

P* → P + hν(7)

**Figure 1:**
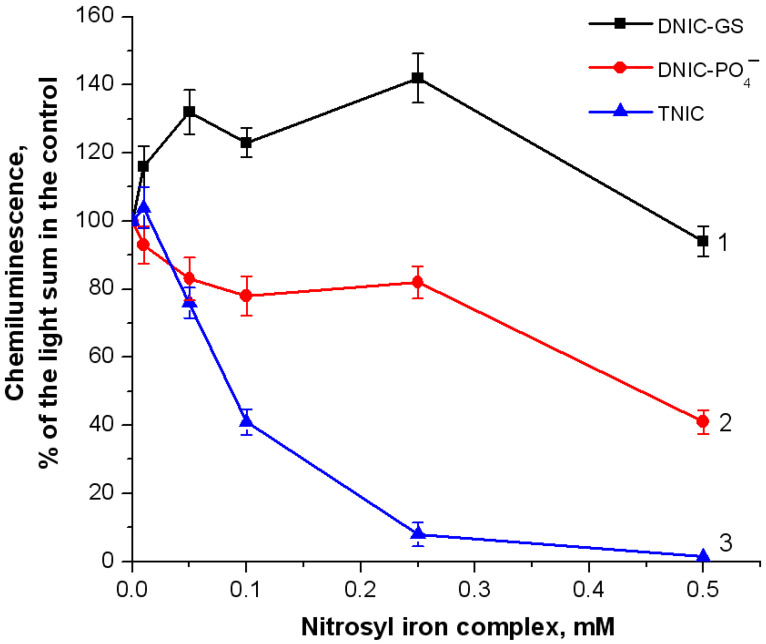
Intensity of luminol-dependent chemiluminescence during the reaction of hemoglobin (Hb) with *tert*-butyl hydroperoxide (*t*-BOOH). Reaction mixture: 0.15 mM metHb in 50 mM K-phosphate buffer (pH of 7.4), 2 mM luminol, and 0.57 mM *t*-BOOH. The chemiluminescence lightsum of 300 s of the control sample without nitrosyl iron complexes was assumed to be 100%. The data are presented as the % of the control lightsum. Complexes added: 1—DNIC-GS, 2—DNIC-PO_4_^−^, and 3—TNIC.

Chemiluminescence intensity is proportional to the rate of photon generation. By interacting with radicals, TNIC inhibited chemiluminescence intensity in a dose-dependent manner. We found that 0.5 mM TNIC completely abolished the radical-induced chemiluminescence, while 0.25 mM TNIC reduced it by approximately 90% (Figure 1, curve 3). DNIC-PO_4_ also suppressed radical production but to a lesser extent. The inhibitory effect of 0.5 mM DNIC-PO_4_^−^ was 60% (Figure 1, curve 2). In contrast, DNIC-GS exerted a prooxidant effect (Figure 1, curve 1) which might be related to the generation of thiyl radicals during glutathione oxidation [38]. Upon interaction with oxygen, these radicals can form glutathione disulfide (GSSG) or thioperoxyl radical (GSOO^•^), as follows:

GS^•^ + O_2_ → GSOO^•^ (8)

GSSG^•−^ + O_2_ → GSSG + O_2_^•−^ (9)

Thiyl radicals are mostly generated during the oxidation of free glutathione present in DNIC-GS preparations. Thiol ligands in the complexes are oxidized without the formation of thiyl radicals [30].

This section provides a concise and precise description of the experimental results, their interpretation, and the experimental conclusions that can be drawn.

Nitrosyl iron complexes can neutralize peroxide radicals since they serve as good donors of NO^•^, which acts as an antioxidant in the LPO system [27,28,29,33]. Nitric oxide can break off LPO chain reactions by interacting with hydroperoxide radicals (k = 1−3 × 10^9^ M^−1^c^−1^) [29], producing organic peroxynitrite (Reaction (10)) and nitrolipids (Reaction (12)), as follows:

ROO^•^ + NO^•^ → ROONO → RO^•^ + NO_2_^•^ (10)

RO^•^ + NO_2_^•^ → RONO_2_ (11)

RO^•^ + NO^•^ → RNO_2_ (12)

**Figure 2:**
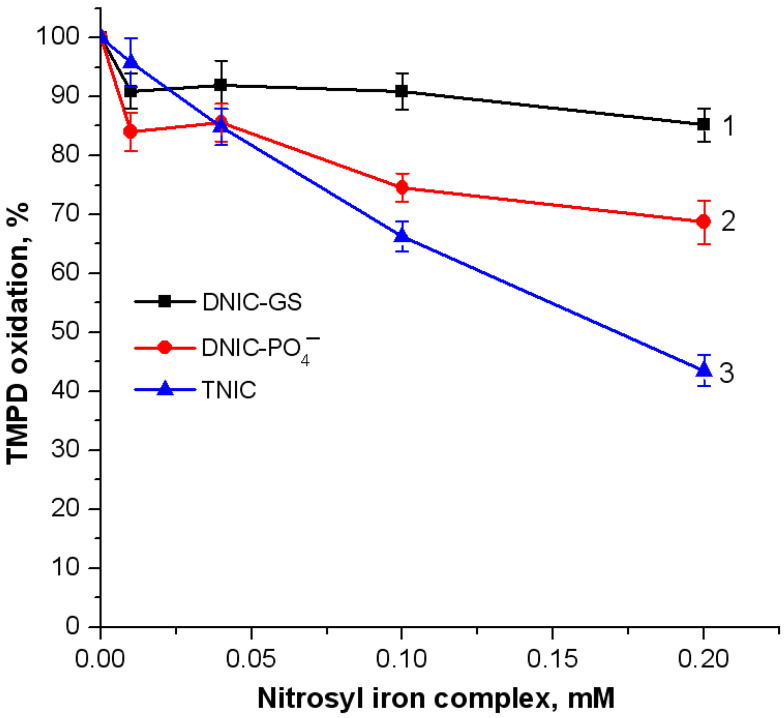
Formation of free radical products in Hb reaction with *t*-BOOH, indicated by *N*,*N*,*N*′,*N*′-tetramethylbenzene-1,4-diamine (TMPD) oxidation. Reaction mixture: 0.03 mM metHb in 50 mM K-phosphate buffer (pH of 7.4), 0.12 mM TMPD, and 0.14 mM *t*-BOOH. The TMPD oxidation level in the control sample without nitrosyl iron complexes was assumed to be 100%. Complexes added: 1—DNIC-GS, 2—DNIC-PO_4_^−^, and 3—TNIC.

The antioxidant and antiradical activities of DNIC-PO_4_^−^, DNIC-GS, and TNIC were also studied in the system of TMPD oxidation by free radical products generated during the reaction of metHb with *t*-BOOH (Figure 2). Similar to the previous model, DNIC-PO_4_^−^ exerted a less pronounced antioxidant effect than TNIC. However, we would like to point ―out that the inhibitory effect of 0.1 mM TNIC was 35%, similar to the effect of 0.2 mM DNIC-PO_4_^−^. DNIC-GS possessed weak antioxidant activity, inhibiting TMPD oxidation by 10–15% (Figure 2, curve 1). 

**Figure 3:**
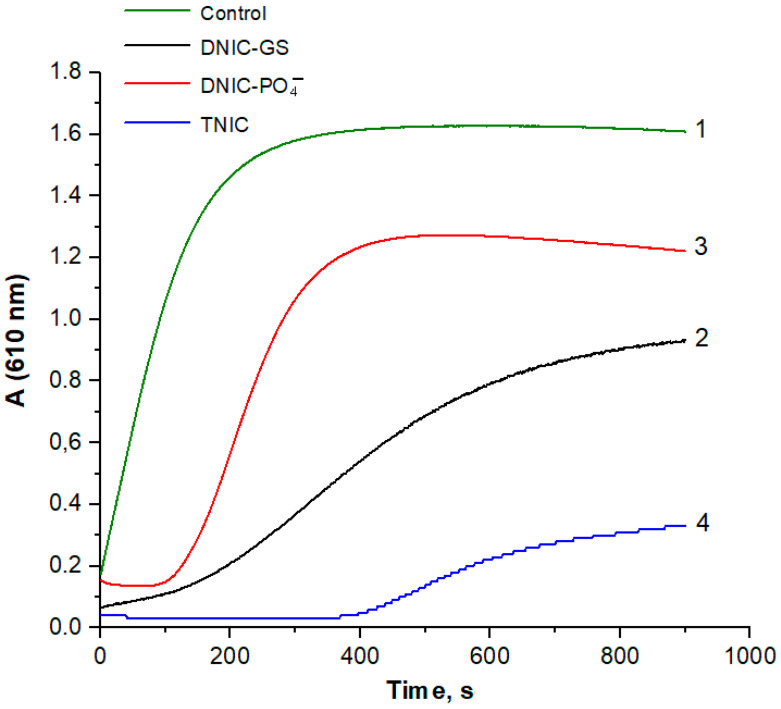
ABTS oxidation (with ABTS^•+^ formation) induced by Hb and *t*-BOOH. Reaction mixture: 0.3 mM metHb in 50 mM K-phosphate buffer (pH of 7.4), 0.7 mM ABTS, and 0.14 mM *t*-BOOH. Nitrosyl iron complexes were added to make final concentrations of 0.25 mM for DNIC-GS (curve 2) and DNIC-PO_4_^−^ (curve 3) and 0.12 mM for TNIC (curve 4). Curve 1—control (without complexes).

The method of estimating the generation of ABTS cation radical (ABTS^•+^) was also used to study the antioxidant effect of nitrosyl iron complexes. Figure 3 shows the kinetics of ABTS^•+^ formation in the reaction system. ABTS^•+^ had an absorption maximum at 743 nm. The addition of nitrosyl iron complexes inhibited ABTS^•+^ production in the metHb/*t*-BOOH system. The lag period is clearly visible on the kinetic curves recorded in the presence of the complexes. TNIC had the longest lag period and the lowest rate of ABTS^•+^ formation (Figure 3, curve 4), which implies that TNIC is capable of ABTS^•+^ reduction. In this reaction system, DNIC-GS exerted a higher antioxidant effect than DNIC-PO_4_^−^. Of note, TNIC concentration in the reaction system was two times lower than that of DNICs. This is because TNIC was a donor of four NO^•^ molecules, whereas DNICs only provided two of them.

### 2.2. Oxidative Modifications of Hemoglobin 

MetHb interaction with hydroperoxides leads to two-electron oxidation with the formation of an oxoferryl intermediate with a cation radical on the porphyrin ring (porphyrin^+•^–Fe^4+^=O). It either destroys the heme group or oxidizes the amino acid residues of the protein [39]. Thus, we investigated the effects of nitrosyl iron complexes on these processes.

**Figure 4:**
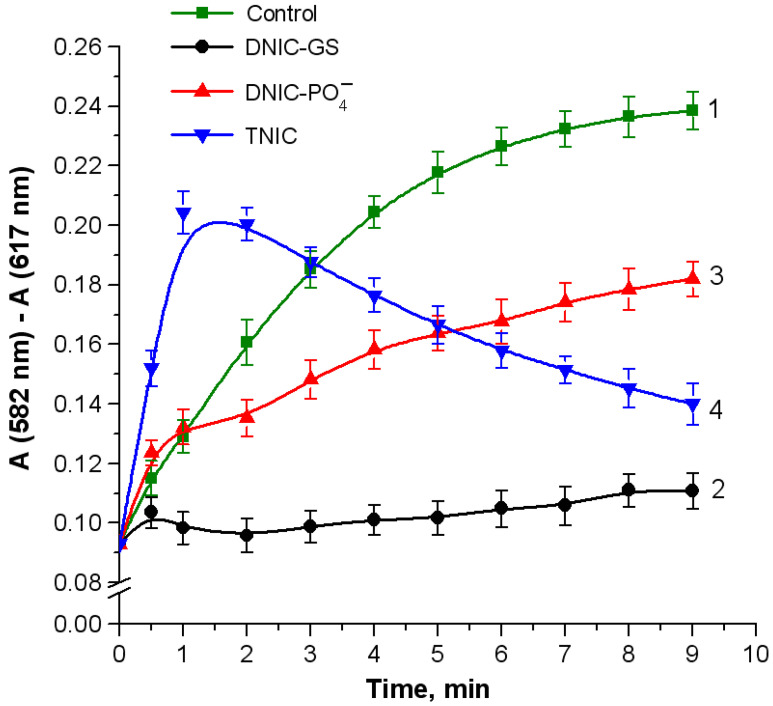
Formation of oxoferrylHb in the Hb reaction with *t*-BOOH. Reaction mixture: 0.03 mM metHb in 50 mM K-phosphate buffer (pH of 7.4) and 0.71 mM *t*-BOOH. Nitrosyl complexes were injected simultaneously with *t*-BOOH. Nitrosyl iron complexes were added to make final concentrations of 0.1 mM for DNIC-GS (curve 2) and DNIC-PO_4_^−^ (curve 3) and 0.05 mM for TNIC (curve 4). Curve 1: control (without complexes).

Nitrosyl iron complexes exerted different effects on oxoferrylHb formation. GS-DNIC possessed the highest inhibitory activity (Figure 4, curve 2), which may be attributed to the action of either NO^•^ or GSH, another component of the complex. The bell-shaped kinetics of TNIC activity (Figure 4, curve 4) is probably related to the formation of nitrosylHb, which has absorption peaks within the recorded region. Nitric oxide can reduce the oxoferryl intermediate to the met-form, followed by the reductive nitrosylation of the heme iron [27], as follows:

Hb-Fe^4+^=O + NO^•^ → Hb-Fe^3+^-ONO → Hb-Fe^3+^ + NO_2_^−^ (13)

OH^−^ Hb-Fe^3+^ + 2NO^•^ → Hb-Fe^2+^-NO + NO_2_^−^ + H^+^ (14)

Hb nitrosylation is known to almost completely inhibit free radical formation [25,40], which implies that Hb-Fe^2+^-NO does not participate in Reactions (1)–(4), but, on the contrary, it restores *t*-BOOH, producing nitrite and metHb, as follows:

Hb-Fe^2+^-NO + *t*-BOOH + H_2_O → Hb-Fe^3+^ + *t*-BOH + HNO_2_ + OH^−^ (15)

According to [25,27], the same mechanism underlies the interaction of *t*-BOOH with metHb and metMb. Therefore, the antioxidant effect of nitrosyl iron complexes in the studied reaction system may also be mediated by Reactions (13)–(15). 

**Figure 5:**
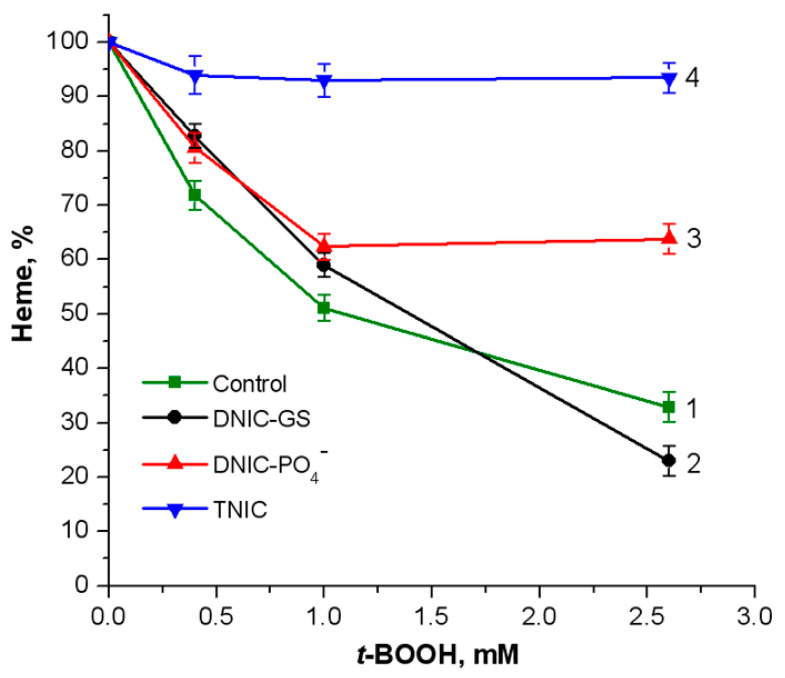
The changes in the content of the native heme group in Hb in the presence of *t*-BOOH. Reaction mixture: 0.15 mM metHb in 50 mM K-phosphate buffer (pH of 7.2) and *t*-BOOH at various concentrations. The heme content in the untreated control sample was assumed to be 100%. Nitrosyl iron complexes were added to make final concentrations of 0.5 mM for DNIC-GS (curve 2) and DNIC-PO_4_^−^ (curve 3) and 0.25 mM for TNIC (curve 4). Curve 1: control (without complexes).

The formation of oxoferrylHb leads to Hb oxidation, accompanied by the degradation of the heme group and release of iron ions, which catalyzes free radical generation in Fenton and Haber–Weiss reactions [38]. Thus, we studied the effect of nitrosyl iron complexes on the destruction of the heme group upon *t*-BOOH action at various concentrations (Figure 5). GS-DNIC (Figure 5, curve 2) had almost no impact on the breakdown of the heme group, while TNIC completely suppressed heme destruction (Figure 5, curve 4). The protective effect of nitrosyl iron complexes on the heme group can be attributed to the ability of NO^•^ released from the complexes to inhibit oxoferrylHb formation. 

Intramolecular porphyrin reduction generates amino acid radicals, mostly tyrosine and tryptophan and, possibly, cysteine residues [39]. The affected residues in the human Hb are αTyr42, αTrp14, βTrp15, βCys93, and the αTyr24–αHis20 complex. The formation of amino acid radicals causes inter-subunit crosslinking, resulting in dimeric and high-molecular-weight protein forms.

**Figure 6:**
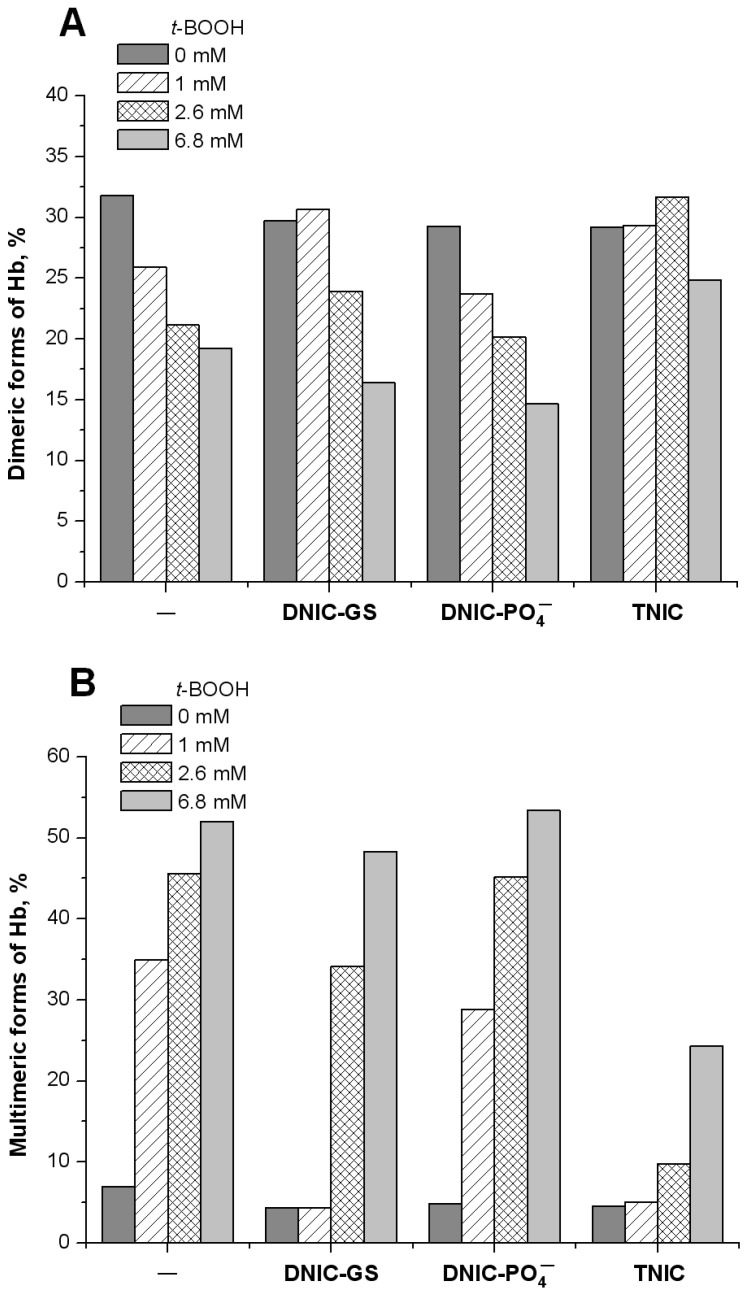
Generation of dimeric (**A**) and multimeric (**B**) Hb forms upon oxidation by various concentrations of *t*-BOOH studied by electrophoresis in 12% SDS-PAGE. The results are presented as percentages of the total protein content, loaded on the gel (band, %). The data shown were obtained by processing electrophoretic images using the “Image Lab Software 6.0.1 (Bio-Rad)” program. The gel is shown in Supplementary Materials.

The aggregates of the intermolecular cross-linked Hb monomers (α- and β-subunits) were detected with electrophoresis in 12% SDS-PAGE. As can be seen in Figure 6, Hb dimer content decreased as *t*-BOOH concentration increased (Figure 6A), except for TNIC. In contrast, the content of multimeric Hb forms increased with the elevation of the oxidant concentration in a dose-dependent manner for all variants (Figure 6B). However, in the case of TNIC, the proportion of Hb-forming multimers was significantly lower for all oxidant concentrations.

The data presented in panels Figure 6A,B imply that Hb dimerization precedes multimer formation. For TNICs, in the presence of 6.8 mM *t*-BOOH, a slight decrease in the dimer fraction was correlated with an increase in high-molecular aggregate content. DNIC-PO_4_^−^ did not inhibit intermolecular Hb crosslinking; GS-DNIC suppressed protein aggregation in the presence of 1 mM or 2.6 mM *t*-BOOH concentrations. Moreover, at a 1 mM concentration, its inhibitory effect was similar to that of TNIC. 

**Figure 7:**
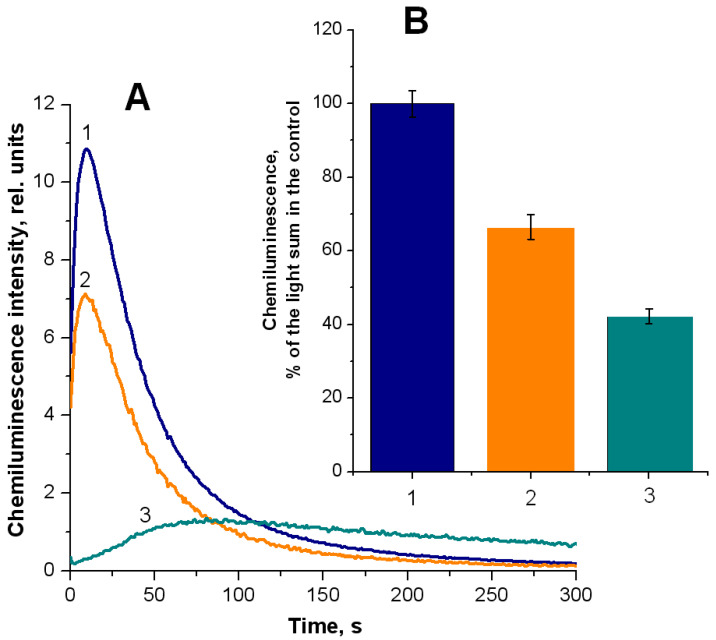
The luminol-dependent chemiluminescence of the products of Hb reaction with *t*-BOOH: the effect of NEM and sodium azide. Reaction mixture: 0.15 mM metHb in 50 mM K-phosphate buffer (pH of 7.4), 2 mM luminol, and 0.57 mM *t*-BOOH. The concentration of NEM was 20 mM, and that of NaN_3_ was 6 mM. (1) metHb, (2) metHb + NEM, and (3) metHb + NEM + NaN_3_. A: kinetics of chemiluminescence intensity. (**B**): column designations correspond to the curves in panel (**A**). The data are presented as the % of chemiluminescence lightsum of the control (1), recorded for 300 s. To obtain NEM-Hb, NEM was added to the Hb solution in a 1:1 molar ratio. The duration of the incubation was 1 h at 20 °C.

The protective effect of nitrosyl iron complexes on Hb, as previously suggested, mostly depends on heme group nitrosylation [25,30,31,35,40]. To confirm this, we carried out an additional experiment with the thiol-specific reagent N-ethylmaleimide (NEM) and the heme ligand sodium azide (NaN_3_). NEM forms a strong thioester bond with the available thiol Hb group (βCys93), with an appearance of NEM-Hb. Azide forms a hexacoordinated low-spin complex with heme iron. Estimating luminol-dependent chemiluminescence showed that adding NEM to the reaction mixture reduces the total chemiluminescence by 34% (Figure 7A, curve 2 and Figure 7B, column 2). Simultaneously introduced NEM and NaN_3_ suppressed chemiluminescence by 58% (Figure 7A, curve 3 and Figure 7B, column 3). 

### 2.3. Oxidation of Linoleic Acid in Hemoglobin/Tert-Butyl Hydroperoxide System 

**Figure 8:**
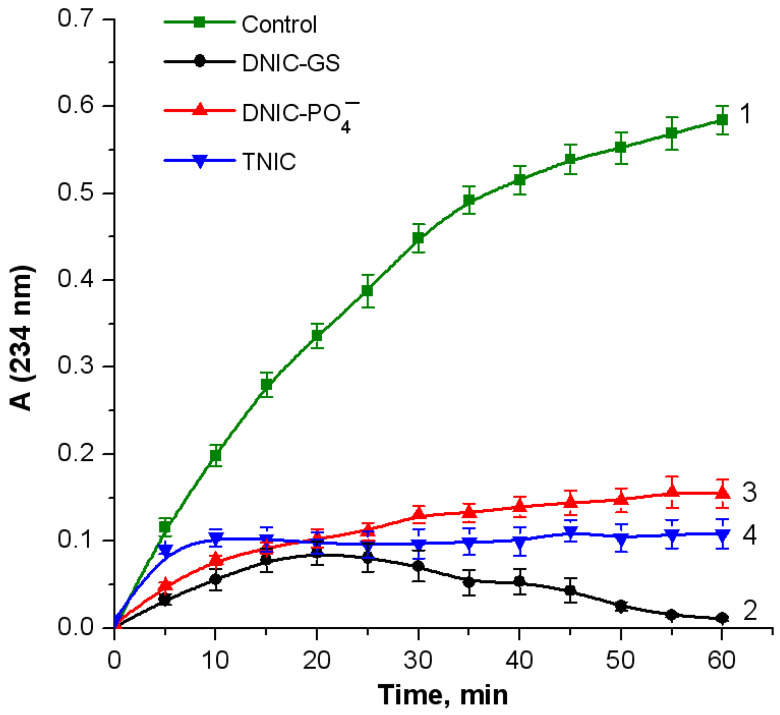
Formation of diene conjugates upon the peroxidation of linoleic acid induced by Hb and *t*-BOOH. Reaction mixture: 0.17 mM linoleic acid, 5.2 µM metHb, and 61 µM *t*-BOOH in 100 mM K-phosphate buffer (pH of 7.4). Nitrosyl iron complexes were added to make final concentrations of 0.05 mM for DNIC-GS (curve 2) and DNIC-PO_4_^−^ (curve 3) and 0.025 mM for TNIC (curve 4). Curve 1: control (without complexes).

OxoferrylHb, which is produced by the interaction of Hb with hydroperoxides, is known to trigger lipid oxidation [38,39], while NO^•^ suppresses free radical lipid oxidation [25,27,28,29]. Thus, we studied the effect of nitrosyl iron complexes on the oxidation of linoleic acid (omega-6-unsaturated fatty acid) in the metHb–*t*-BOOH system. The level of fatty acid oxidation was estimated by the formation of diene conjugates, the primary oxidation products [41]. All the complexes studied had a pronounced inhibitory effect on arachidonic acid oxidation. Fatty acid oxidation was almost completely abolished after 10 min, whereas in the control sample, it was registered for the entire measurement period (Figure 8). Moreover, DNIC-GS exerted a higher antioxidant activity compared to DNIC-PO_4_^−^ and TNIC. These results might be related to the suppressed prooxidant effects of hemoproteins by NO^•^, released from the complexes during oxoferryl heme reduction (Reaction (13)) and heme iron nitrosylation (Reaction (14)). Moreover, NO^•^ can inhibit LPO reactions by interacting with alkoxyl and alkylperoxyl lipid radicals (Reactions (11) and (12)).

## 3. Discussion

In this work, we scrutinized the nitrosyl iron complexes: DNICs with glutathione ligands and TNIC with thiosulfate ligands (Na_2_[Fe_2_(S_2_O_3_)_2_(NO)_4_]·4H_2_O). We chose these compounds since they are generally extensively studied, with proven pharmacological activity; we compared their properties with DNICs with phosphate ligands. This complex is characterized by low stability, which makes it convenient to utilize it as a donor of the [Fe(NO)_2_]^+^ fragment for further synthesis of peptides or proteins associated with DNICs. TNIC with thiosulfate can also be used to produce anionic DNICs with S-donor ligands [12].

DNICs with glutathione ligands are generated in the organism by the interaction of NO^•^ with a labile iron pool (LIP). GSH is considered to serve as the main LIP ligand since it is present in the cell at high concentrations (2–10 mM) and has a high affinity for Fe^2+^ (k = 1.3 ·10^5^ M^−1^s^−1^) [42]. LIP is a main target for NO^•^ [43,44]. The reaction rate between LIP and NO^•^ is higher compared to Fe^2+^ dissolved in water. The product of this reaction is a mononitrosyl iron complex (L▪▪▪Fe^3+^NO) [45], which generates DNIC (DNIC-RS) while reacting with thiols. DNIC-RS is one of the most common NO^•^ metabolites that deposit and translocate this mediator in cells [46,47,48].

DNIC formation during the LIP reaction with NO^•^ has been shown in macrophages [43]. In erythrocytes, LIP is involved in DNIC generation as well. In this case, NO^•^ is produced by the erythrocyte NO synthase (eNOS) [49] and the nitrite reductase in the reaction catalyzed by Hb [50].

DNICs and S-nitrosothiols form a self-regulating system, together with iron ions, thiols, and nitric oxide. This system can be a donor of free NO^•^ molecules as well as of nitrosonium ions (NO^+^) and dinitrosyl iron fragment [Fe(NO)_2_]^+^, which exhibit high affinity for thiols [19]. DNICs exert a variety of biological effects, acting as signaling agents and regulating gene expression, apoptosis, enzymatic activity, and iron metabolism. DNICs with thiol ligands have high antihypertensive and vasodilatory effects [18]. A prolonged vasodilatory effect of DNIC-RS is thought to depend on S-nitrosothiols (RSNO) or S-nitrothiols (RSNO_2_) [48]. DNIC-RS complexes inhibit platelet aggregation, facilitate the repair of tissue, and promote skin wound healing [48]. Moreover, DNICs associated with Hb and albumin are crucial for preserving and translocating NO^•^ in human and animal circulatory systems [51].

It is currently of great medical importance to devise dual-acting drugs that synthesize antioxidant and unrelated therapeutic effects. Since nitrosyl iron complexes may fulfil both criteria, we investigated their antioxidant effects. Unfortunately, standardized methods for determining the antioxidant properties of chemical compounds have not been developed yet. Since the use of a single method or experimental model does not provide complete information on the antioxidant and antiradical properties of the compound, we used several approaches to evaluate different aspects of antioxidant properties.

There are three main mechanisms underlying the inhibition of free radical oxidation: (1) electron transfer, (2) proton transfer coupled with the electron one, and (3) the complexation of metal ions of variable valence (Zn^2+^, Fe^2+^, and Cu^2+^). The antioxidant effect of nitrosyl iron complexes is mediated by different molecular mechanisms, primarily provided by mechanism number 1, since NO^•^ acts as an electron donor for many radicals.

NO^•^ has been shown to inhibit LPO in various experimental systems. The main mechanism of its action is associated with the reaction of NO^•^ with a peroxyl radical (Reaction (10)); the concentration of the latter determines the rate of the self-propagation of the LPO reaction [29]. This radical can extract bis-allyl hydrogen from a neighboring fatty acid, forming lipid hydroperoxide and the second lipid radical (Reaction (16)). The latter subsequently reacts with oxygen, regenerating the peroxyl radical (Reaction (17)). These reactions represent the stages of the development of the LPO chain:

ROO^•^ + RH → ROOH + R^•^ (16)

R^•^ + O_2_ → ROO^•^ + R (17)

The second mechanism of LPO inhibition by nitrosyl iron complexes is the prevention of initial lipid oxidation. This can be mediated by both the nitrosylation of heme proteins [25,27,28,41] and Fe^2+^ chelation. Incorporating Fe^2+^ ions into DNICs makes them inaccessible to Fenton-type reactions [46] that serve as a source of hydroxyl radicals. Moreover, introducing NO^•^ into complexes slows down the generation of a peroxynitrite.

The ligands incorporated into the complexes can act as antioxidants as well. DNIC-GS contains glutathione, a powerful antioxidant acting as the first line of defense against free radicals in the organism. However, in the presence of metal ions, thiols can acquire prooxidant properties [52].

We cannot rule out the possibility that the nitrosyl iron group in DNICs ([Fe(NO)_2_]^+^) can react with LPO free radical intermediates. The reaction between nitrosyl complexes of iron or copper and molecular oxygen or H_2_O_2_ produces intermediates containing peroxynitrite bound to metal ions [53,54]. It seems very likely that DNICs catalyze the formation of organic peroxynitrite derivatives (ROONOs) and their further degradation, forming non-toxic products. The interaction of thiol-containing DNICs with peroxynitrite results in an intermediate containing an iron-bound peroxynitrite, namely, (GS)_2_-Fe^+^-(ONOO)(NO), which is further decomposed. In the course of the reaction, it releases non-radical products or is involved in the nitration of biomolecules [31,55]. Therefore, DNICs, acting as catalysts of peroxynitrite decomposition, can perform multiple roles. They can scavenge powerful prooxidants, initiate LPO, and oxidize fat-soluble antioxidants and proteins [3,29].

During the Hb reaction with H_2_O_2_, the βCys93 amino acid residue is primarily oxidized to the cysteine radical [56]. Other amino acids of the β-chain are oxidized following the depletion of βCys93. A study on the role of βCys93 in the metabolism of reactive compounds revealed that this residue was a redox-sensitive [57] and an allosterically controlled antioxidant [58]. Acting as NO^+^ donors, DNIC-RS can thus induce the S-nitrosylation of thiol-containing compounds with a high and low molecular weight [44,48]. Nitrosylating the Hb redox-sensitive cysteine β93Cys by the DNIC decay products or NO^•^ oxidation metabolites (N_2_O_3_ and ONOO^−^) can be considered as a reversible non-enzymatic posttranslational modification, protecting this residue from oxidation and preventing Hb inactivation.

Our data indicate that DNICs and TNICs exhibit the antioxidant effect in the system modeling of Hb oxidation by organic hydroperoxide. This is consistent with our previous results, which showed that DNICs associated with the Hb cysteine residue or glutathione effectively prevent Hb oxidation [31,35]. The antioxidant action of DNICs with thiol ligands was also demonstrated in other model systems [30,31,32,35,59]. 

DNIC formation mitigates oxidative stress in cells [34,60]. We demonstrated that DNIC-GS protected erythrocytes against HOCl-induced hemolysis [32]. DNIC-GS was shown to reduce the production of reactive oxygen species using the model of isolated rat hearts subjected to cardioplegic arrest and reperfusion [11]. The cytoprotective effect of DNICs might be mediated by its direct antioxidant effect as well as by other mechanisms. For instance, DNICs with S-donor ligands, such as thiocarbamide and its derivatives, uncoupled oxidative phosphorylation in mitochondria, causing the leakage of protons across the mitochondrial membrane [61]. This effect increased the viability of human lung fibroblasts and rat cardiomyocytes in the presence of doxorubicin [61].

The balance of the antioxidant/prooxidant properties of nitrosyl iron complexes depends on their chemical and electronic structure, concentration, and duration of NO^•^ release. The prooxidant properties of certain complexes are employed for oncotherapy [12,13,20,26]. For example, the interaction of DNIC with N-ethylthiourea on HeLa tumor cells induces the accumulation of reactive oxygen and nitrogen species, thus activating proapoptotic enzymes [13].

The limited use of nitrosyl iron complexes in biomedicine is due to several concerns; the main one is their low stability, toxicity of the decay products, e.g., Fe^2+^, and explosive kinetics of NO^•^ release. Research is currently focused on designing stable systems to deliver complexes with controlled NO^•^ release [14,17]. Utilizing these systems is especially important for the treatment of oncological diseases. Such promising complexes include the nitrosyl iron complex with thiourea and thiosulfate ligands [Fe(SC(NH_2_)_2_)_2_(NO)_2_]_2_[Fe_2_(S_2_O_3_)_2_(NO)_4_], which is decomposed under aerobic conditions to form the Fe(Cys34)(His39)(NO)(NO_2_) complex bound to bovine serum albumin [62].

## 4. Materials and Methods

### 4.1. Materials and Reagents 

The following reagents were used in this work: methemoglobin (metHb) from bovine erythrocytes, 4-(2-hydroxyethyl)piperazine-1-ethanesulfonic acid (HEPES), 5-amino-2,3-dihydrophthalazine-1,4-dione (luminol), reduced glutathione (GSH), N-ethylmaleimide (NEM), *tert*-butyl hydroperoxide (*t*-BOOH), dimethylsulfoxide (DMSO), 2,2′-azino-bis(3-ethylbenzothiazoline-6-sulfonic acid) (ABTS), polyacrylamide (PAA), glycerol, glycine, linoleic acid, pyridine, *N*,*N*,*N*′,*N*′-tetramethylbenzene-1,4-diamine (TMPD), Coomassie brilliant blue R-250, dithiothreitol (DTT), 2,4-dinitrophenylhydrazine (DNPH), tris(hydroxymethyl)aminomethane (Tris), sodium dodecyl sulfate (SDS), sodium dithionite (Na_2_S_2_O_4_·2H_2_O), NaNO_2_, ferrous sulfate (FeSO_4_·7H_2_O), KH_2_PO_4_, K_2_HPO_4_ (“Sigma-Aldrich” (St. Louis, MO, USA)), and 4-hydroxy-(2,2,6,6-tetramethylpiperidin-1-yl)oxyl (4-hydroxy-TEMPO) (“Oxis” (Portland, OR, USA)).

Paramagnetic phosphate-bound DNICs (DNIC-PO_4_^−^) were synthesized as described in [31]. In brief, gaseous NO^•^ was passed through the FeSO_4_ solution (5 mM) in 100 mM K-Na phosphate buffer (pH of 7.0) in the Thunberg tube. Generated DNIC incorporated all the introduced iron. DNICs with glutathione (DNIC-GS) ligands were obtained by adding the glutathione solution to phosphate DNICs at a molar ratio of 1:2. The DNIC concentration was calculated from the integral intensity of the EPR signal from these complexes, using the 4-hydroxy-TEMPO spin label as a standard. DNIC preparations were stored at −70 °C.

The Na_2_[Fe_2_(S_2_O_3_)_2_(NO)_4_]·4H_2_O complex was synthesized as follows. Gaseous NO^•^ was passed through the anaerobic aqueous solution containing the mixture of FeSO_4_·7H_2_O and Na_2_S_2_O_3_·5H_2_O at room temperature. The resulting complex was crystallized at +4 to +8 °C. 

### 4.2. Measurement of Luminol-Dependent Chemiluminescence 

The generation of free radical intermediates during the reaction of Hb with *t*-BOOH was assessed using chemiluminescence from luminol, which was used as an activator registered by the Lum-100 chemiluminescence analyzer (“DISoft”, Moscow, Russia), starting 3 s after mixing the components of the reaction mixture. During kinetics registration, the mixture was continuously stirred and kept at 37 °C. The total chemiluminescence from the readings recorded within 300 s was used to process the experimental curves. It was calculated from the area under a kinetic curve and used for chemiluminescence quantification. The reaction mixture contained 0.15 mM metHb in 50 mM K, Na-phosphate buffer (pH of 7.4), 2 mM luminol, and 0.57 mM *t*-BOOH. 

### 4.3. Obtaining and Registering OxoferrylHb 

The effect of nitrosyl iron complexes on the formation of oxoferrylHb (Hb-Fe^4+^=O) was studied in a system containing *t*-BOOH. Briefly, 0.71 mM *t*-BOOH was added to 0.03 mM metHb in 50 mM K-phosphate buffer (pH of 7.4) at a ratio of 6:1 per Hb subunit. Nitrosyl iron complexes were introduced simultaneously with *t*-BOOH at a concentration of 0.4 mM, which corresponds to the 3:1 protein/complex molar ratio per protein subunit. The accumulation of Hb-Fe^4+^=O was monitored by the difference between absorption values at 582 nm and 617 nm (the isobestic point nearest to the 582 nm absorption peak). 

### 4.4. Formation of the Products of Hemoglobin Oxidation in the Reaction with TMPD 

ROS production during the reaction of metHb and *t*-BOOH was measured in the presence of TMPD [63]. t-BOOH was added to the 0.03 mM metHb solution in 50 mM K-phosphate buffer (pH of 7.4) with 0.12 mM TMPD to a final *t*-BOOH concentration of 0.14 mM. Optical absorption was measured at 610 nm. Oxidant production was estimated by the area under the TMPD oxidation curve registered for five minutes. 

### 4.5. Antioxidant Activity Measurement with ABTS 

The antioxidant effect of nitrosyl iron complexes was also studied by estimating the generation of ABTS cation radical (ABTS^•+^) [64]. A blue-green radical ABTS^•+^ is produced in the reaction system containing metHb and *t*-BOOH. Adding nitrosyl iron complexes reduces the cation radical to ABTS, which is accompanied by the discoloration of a solution [65]. The course of the reaction was monitored at 734 nm. The reaction mixture contained 0.03 mM metHb in 50 mM K-phosphate buffer (pH of 7.4), 0.7 mM ABTS, and 0.14 mM *t*-BOOH. 

### 4.6. Measurement of Linoleic Acid Diene Conjugates 

The content of diene conjugates and the primary products of linoleic acid oxidation induced by metHb reaction with *t*-BOOH was calculated from the optical absorption measured at 234 nm. The reaction mixture contained 0.17 mM linoleic acid, 5.2 µM MetHb, and 61 µM *t*-BOOH in 100 mM K-phosphate buffer (pH of 7.4). The kinetics was recorded for 60 min at 37 °C by the Cary 300 Bio spectrophotometer (“VarianBio”, Naples, FL, USA).

### 4.7. Determination of the Content of the Heme Group 

Heme content in the Hb solution was measured following the pyridine hemochrome method [66], with modifications. Briefly, 135 µL of water and 450 µL of 30% alkaline pyridine solution were added to 15 µL of Hb solution. Immediately prior to the measurement, the solution was reduced with sodium dithionite. The optical absorption of the reduced heme complex with pyridine was measured at 556 and 539 nm. The reaction mixture contained 0.15 mM metHb in 50 mM K-phosphate buffer (pH of 7.4). The incubation with different *t*-BOOH concentrations (0.4, 1, and 2.6 mM) was carried out for 30 min. 

### 4.8. SDS-PAGE Electrophoresis 

Electrophoresis was performed in 12% polyacrylamide gel (150 × 150 × 1 mm) according to the Laemmli method [67]. The sample buffer was added to protein solutions at a ratio of 1:1, heated for 5 min at 95 °C, and loaded onto a gel. The 0.1 M Tris-HCl buffer (pH of 6.8) sample contained 4% SDS, 0.2% bromophenol blue, and 20% glycerol. A total of 10 mL of protein solution was loaded onto a gel. Then, 0.2 M Tris-glycine buffer (pH of 8.3) containing 0.1% SDS was used as an electrode buffer. Electrophoresis was carried out at 4 °C, I = 50 mA, and U = 150 V. (Elf-4 power supply, “DNA-Technology”, Moscow, Russia). After protein separation, the gel was fixed and stained using the Coomassie brilliant blue R-250 solution. 

### 4.9. Spectrophotometric Studies

Hb optical absorption spectra were recorded using the Cary 300 spectrophotometer (“VarianBio”, Naples, FL, USA) at room temperature in a 1 cm optical path length cuvette at a 600 nm/min scanning speed. 

### 4.10. Statistical Analysis

The measurements were performed with at least three replicates for each sample. The statistical data were processed based on 3–4 analytical repetitions. The data are presented as the mean ± standard deviation. The ANOVA statistical model and Student’s *t*-test were used for the data analysis. The differences were considered to be statistically significant when the *p*-value was less than 0.05.

## 5. Conclusions

We performed a comparative study of the antioxidant and antiradical effects of nitrosyl iron complexes (DNICs and TNICs) with various ligands in the model system of Hb oxidation by *tert*-butyl hydroperoxide. To scrutinize the antioxidant properties of the studied compounds, we employed various methods, which allowed us to evaluate their antioxidant as well as prooxidant effects. The obtained data imply that TNICs have a pronounced antioxidant effect in all studied Hb-containing systems, while DNIC-GS functions either as an antioxidant or as a prooxidant under different conditions. The prooxidant effect of DNIC-GS comes from thiol ligands, which are oxidized, generating thiyl radicals. The prooxidant effect of DNIC-PO_4_^−^ is probably caused by their low stability and a subsequent release of Fe^2+^, which catalyzes ^•^OH formation.

The stronger antioxidant properties of TNICs compared to the studied DNIC types can be related to their high ability to generate NO^•^, the lower iron content per 1 mol of NO^•^ of the complex, and the absence of a prooxidant action of the decay products. The high NO-generating activity of TNICs is mediated by the mononuclear nitrosyl intermediates produced upon the oxidation of the complexes under aerobic conditions [24].

Many diseases are accompanied by oxidative and nitrosative stress. Carbonyl stress often affects cardiovascular and blood systems, including erythrocytes and Hb, e.g., under diabetes and related diseases [68]. Our results indicate that TNICs and DNIC-GS may be useful for the development of medicine used in clinical practice as vasodilators and cardioprotective drugs thanks to their antioxidant and antiradical properties.

## Data Availability

The data presented in this study are available in the article.

## Supplementary Materials

The supporting information can be downloaded at: https://www.mdpi.com/article/10.3390/ijms25137194/s1.
