# Supplementary material for: Effects of Nitrosyl Iron Complexes with Thiol, Phosphate, and Thiosulfate Ligands on Hemoglobin
Source: Int J Mol Sci. 2024 Jun 29;25(13):7194. doi: 10.3390/ijms25137194 (PMC11241041; doi:10.3390/ijms25137194)
Supplement: Supplementary file 1 [file ijms-25-07194-s001.zip › ijms-3028077-supplementary.pdf]

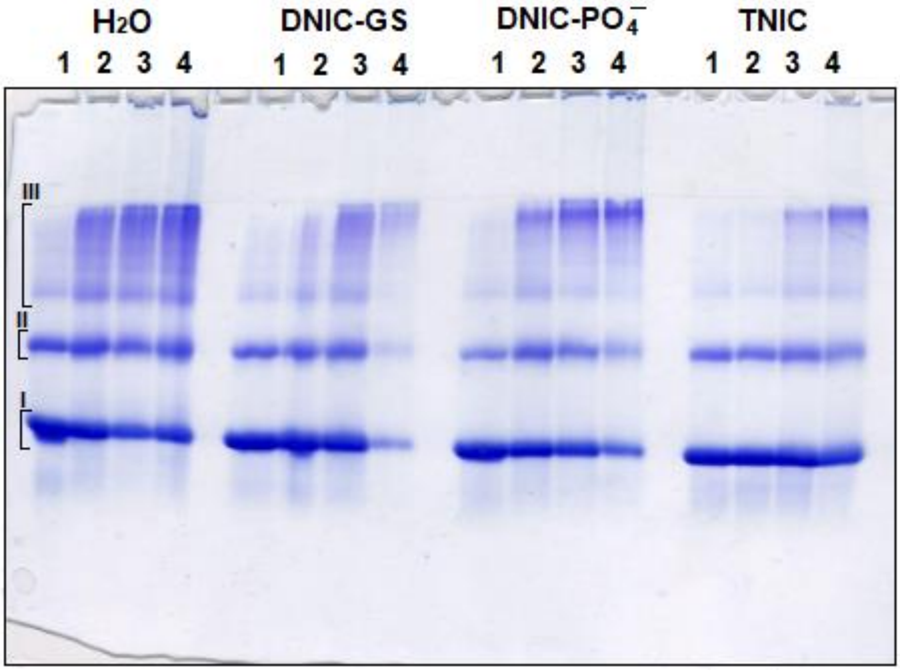

An example of SDS-PAGE electrophoresis in 12% PAGE of metHb treated with different amounts of *t*-BOOH and protected by nitrosyl iron complexes.

1 – 0 mM *t*-BOOH; 2 - 1 mM *t*-BOOH; 3 – 2.6 mM t-BOOH; 4 – 6.8 mM *t*-BOOH.

I – Mixture of dissociated α- and β- Hb monomers (subunits) (~16 kDa); II – Hb dimers (~32 kDa); III – Hb multimers (>32 kDa) including tetramers (~64 kDa).
